# Gut Microbial Profile Is Associated With the Severity of Social Impairment and IQ Performance in Children With Autism Spectrum Disorder

**DOI:** 10.3389/fpsyt.2021.789864

**Published:** 2021-12-17

**Authors:** Zilin Chen, Kai Shi, Xin Liu, Yuan Dai, Yuqi Liu, Lingli Zhang, Xiujuan Du, Tailin Zhu, Juehua Yu, Shuanfeng Fang, Fei Li

**Affiliations:** ^1^Department of Developmental and Behavioral Pediatric & Child Primary Care, Brain and Behavioral Research Unit of Shanghai Institute for Pediatric Research and MOE-Shanghai Key Laboratory for Children's Environmental Health, Xinhua Hospital, Shanghai Jiao Tong University School of Medicine, Shanghai, China; ^2^Institute of Science and Technology for Brain-Inspired Intelligence, Fudan University, Shanghai, China; ^3^Centre for Experimental Studies and Research, First Affiliated Hospital of Kunming Medical University, Kunming, China; ^4^Department of Child Health Care, Children's Hospital Affiliated to Zhengzhou University, Zhengzhou, China

**Keywords:** autism, gut microbiota, 16S rRNA, developmental delay/intellectual disability, intelligence quotient

## Abstract

**Background and Objective:** Autism spectrum disorder (ASD) refers to a heterogeneous set of neurodevelopmental disorders with diverse symptom severity and comorbidities. Although alterations in gut microbiota have been reported in individuals with ASD, it remains unclear whether certain microbial pattern is linked to specific symptom or comorbidity in ASD. We aimed to investigate the associations between gut microbiota and the severity of social impairment and cognitive functioning in children with ASD.

**Methods:** A total of 261 age-matched children, including 138 children diagnosed with ASD, 63 with developmental delay or intellectual disability (DD/ID), and 60 typically developing (TD) children, were enrolled from the Shanghai Xinhua Registry. The children with ASD were further classified into two subgroups: 76 children diagnosed with ASD and developmental disorder (ASD+DD) and 62 with ASD only (ASD-only). The gut microbiome of all children was profiled and evaluated by 16S ribosomal RNA sequencing.

**Results:** The gut microbial analyses demonstrated an altered microbial community structure in children with ASD. The alpha diversity indices of the ASD+DD and ASD-only subgroups were significantly lower than the DD/ID or TD groups. At the genus level, we observed a decrease in the relative abundance of *Prevotella*. Simultaneously, *Bacteroides* and *Faecalibacterium* were significantly increased in ASD compared with DD/ID and TD participants. There was a clear correlation between alpha diversity and the Childhood Autism Rating Scale (CARS) total score for all participants, and this correlation was independent of IQ performance. Similar correlations with the CARS total score were observed for genera *Bacteroides, Faecalibacterium*, and *Oscillospira*. However, there was no single genus significantly associated with IQ in all participants.

**Conclusions:** Specific alterations in bacterial taxonomic composition and associations with the severity of social impairment and IQ performance were observed in children with ASD or ASD subgroups, when compared with DD/ID or TD groups. These results illustrate that gut microbiota may serve as a promising biomarker for ASD symptoms. Nevertheless, further investigations are warranted.

## Introduction

Autism spectrum disorders (ASDs) are a set of neurodevelopmental behavioral disorders associated with impaired social communication and restricted, repetitive behaviors in early childhood (1). A significant increase in the overall prevalence of ASD has been observed, which is now estimated at 1 in 54 in the United States (2).

In addition to atypical behavioral characteristics, such as impaired social cognition, social perception, and executive dysfunction, children diagnosed with ASD often experience other comorbidities (3), most commonly, developmental delay or intellectual disability (DD/ID). Approximately 1–3% of the global population has intellectual disabilities with below-average general intellectual function and limitations in adaptive functioning (intelligence quotient <70) according to World Health Organization statistics. In contrast, ~70% of individuals diagnosed with ASD have some form of DD/ID (4). It is widely accepted that the clinical manifestations of ASD are highly heterogeneous in terms of symptom severity and cognitive function, ranging from non-verbal children with severe intellectual impairments to high-functioning individuals with no developmental delay and normal or superior IQ (5–7). Emerging clinical and epidemiological studies have shown remarkable differences in clinical manifestations, prognosis, and support requirements for individuals diagnosed with ASD and developmental disorder (ASD+DD) vs. ASD only. Compared to the children with ASD-only, those diagnosed with ASD+DD often exhibit obvious clinical features, including low maternal serotonin levels, low paternal IQ, and increased challenging behaviors (8, 9). Moreover, the children with ASD+DD diagnosis and relatively low IQ scores often exhibit higher levels of stereotypical behavior, self-harming, and sleep problems than individuals with ASD-only (10–12). Large-scale genome sequencing studies also suggest that the disruption rate of ASD-related genes is significantly higher in the presence of comorbid DD/ID (5, 13).

Recent studies indicate that children with ASD have an increased prevalence of gastrointestinal (GI) symptoms, including abdominal distension, constipation, and diarrhea, suggesting changes in intestinal physiology, compared to children with other developmental disorders or those who are typically developing (TD) and healthy sibling pairs (14). Further studies show that gut microbiota may play a significant role in human physiology and pathology, and the prominent GI symptoms in ASD individuals (15). The human gut microbiota is a complex population of 10^14^ microbes, including bacteria and fungi, whose genomes (microbiome) contain at least 100 times as many genes as the human genome (16). It is now well-accepted that the gut microbiota contributes to normal metabolism and immune homeostasis and could also modulate central nervous system activities and behaviors through neural, immune, and endocrine pathways (17). Evidence linking ASD and abnormalities in gut microbial function has been accumulating. Alterations in the composition of gut microbiota community structure have been reported in individuals with ASD and animal models of autism (18, 19). In human populations, the stool of children with ASD was shown with significant decreases in *Bifidobacterium, Prevotella, Blautia*, and *Dialister*, as well as significant increases in *Clostridium* compared with TD or healthy sibling pairs (20, 21). More recently, transplantation of gut microbiota from human donors with ASD or neurotypical controls into germ-free mice revealed that colonization with ASD microbiota is sufficient to induce hallmark behaviors of ASD (22). However, it remains unclear whether microbial patterns contribute to specific symptoms or comorbid medical problems in patients with ASD. In particular, there have been no studies focused on how DD/ID affects the gut microbiota of children with ASD.

Therefore, the present study aimed to investigate the gut microbiota of children with ASD in large sample size, and to clarify the association between the gut microbial spectrum and clinical manifestations (social impairment and intellectual disability) in children with ASD, by recruiting two groups of control participants, including TD and DD/ID. Furthermore, we classified children with ASD into two subgroups (ASD+DD and ASD-only), allowing us to ascertain how developmental delay or intellectual disability could influence the gut microbiota profile in a non-genetic situation and pinpoint potential microbiota-associated mechanisms and signaling pathways for related behavioral deficits in ASD.

## Methods

### Ethics

The study was approved by the Ethical Committee of Shanghai Jiao Tong University School of Medicine Affiliated Xinhua Hospital and conducted following the relevant guidelines and regulations. Written informed consent was obtained from the children and their parents following the Declaration of Helsinki. We adhered to standard biosecurity and institutional safety procedures in this study.

### Selection and Description of Participants

**Figure 1:**
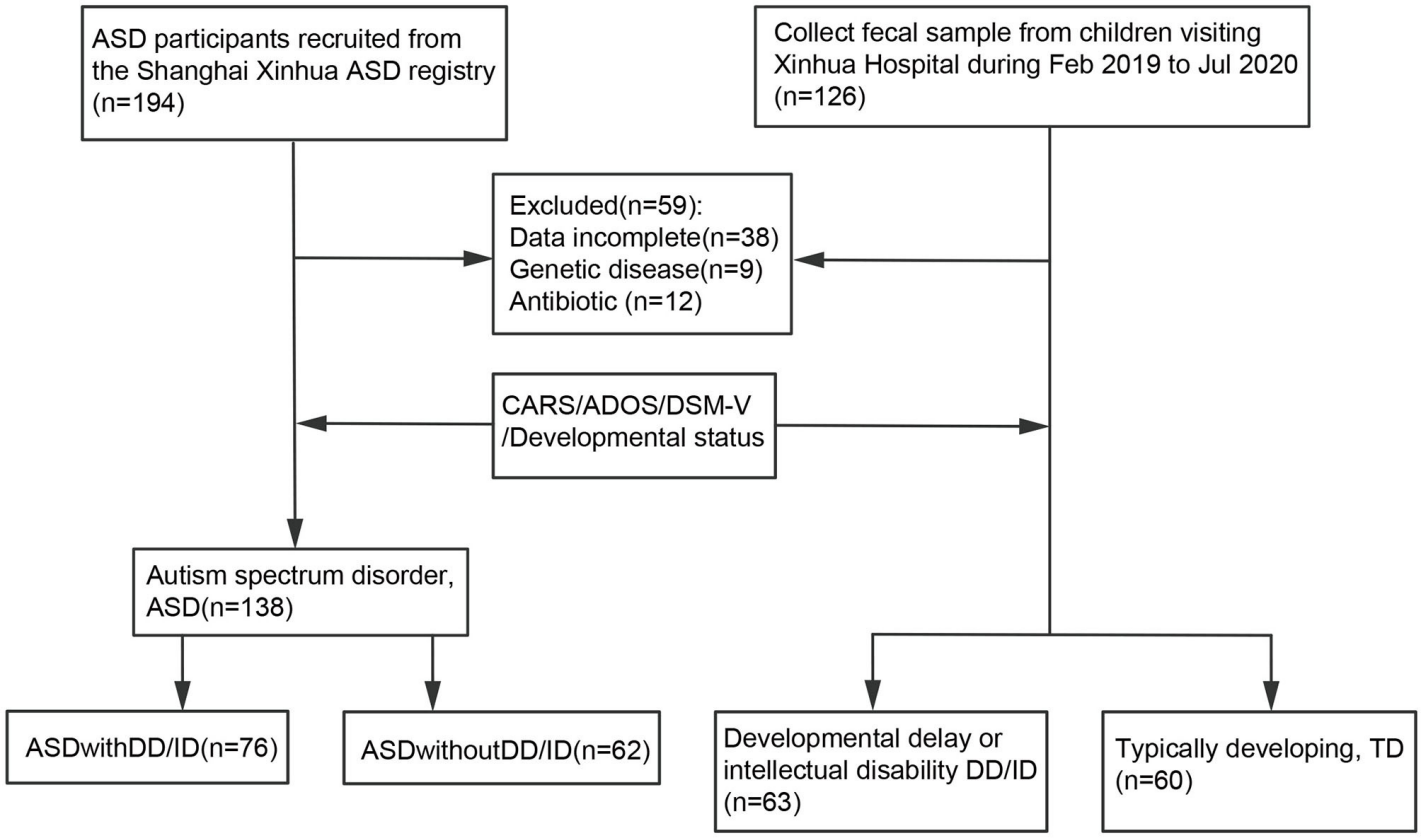
Flowchart of the study design.

ASD subjects were enrolled from the Shanghai Xinhua ASD Registry in Shanghai, China. The subgroup of ASD-only was screened to ensure the children matched the general population. Only children with an agreed clinical diagnosis using the Diagnostic and Statistical Manual of Mental Disorders (Fifth Edition, DSM-V) were included in the ASD group (*n* = 138, 117 males and 21 females) (Figure 1). The diagnosis was confirmed by scores above the cut-off points of two tests: the Autism Diagnostic Observation Schedule (ADOS) and Childhood Autism Rating Scale (CARS). The ASD group was also classified according to whether the children presented with DD/ID [developmental quotient (DQ) <75 and/or intelligence quotient (IQ) <70] (23). Additionally, a group with developmental delay or intellectual disability (DD/ID) and a group of TD children were selected and matched with same-age children visiting the Outpatient Clinic of the Department of Developmental Behavioral Pediatrics and Children's Primary Healthcare at Xinhua Hospital, Shanghai, China from January to December 2020. Those who did not meet the criteria for ASD but were developmentally delayed with DQ <75 were assessed using the Gesell development scales. Those with an IQ <70 were assessed using the WISC-R or WPPSI (Wechsler Intelligence Scale for children). These children were then included in the DD/ID group (*n* = 63, 46 males and 17 females) (Figure 1). Thus, children in the DD/ID group scored below the ASD cut-off score on the ADOS and <30 on the CARS. The clinical signs and absence of illness in these children were confirmed, and all of them underwent an evaluation consisting of the ADOS, CARS, and intellectual assessment. Participants were excluded if they had undergone any antibiotic treatment for 1 month before the sample collection or presented with a diagnosis of chromosomal abnormality or other known neurological, metabolic, or genetic diagnoses.

### Behavior Scale and Gastrointestinal Symptoms Scale Assessment

The CARS is made up of 15 items rated on a 7-point scale from 1 to 4. Higher scores are associated with a higher level of impairment, and scores below 30 do not indicate ASD. We categorized these items into three CARS domains: Social impairment, Negative emotionality, and Distorted sensory response (24). Social impairment is the sum of 11 subscale scores, including cars1, cars2, cars4, cars5 cars10, cars11, cars12, cars13, cars14, and cars15. Negative emotionality is the sum of 3 subscale scores, including cars3, cars6, and cars10. Distorted sensory response is the sum of 3 subscale scores, including cars7, cars8, and cars9. ADOS was used as a supplement to diagnose ASD, and comprises modules tailored to the language level and age of the individual. Four sub-items for assessment of Social interaction, Communication, Play, and Imaginative use of materials were used (25).

An evaluation of GI symptoms was completed by 247 participants (ASD:137, DD/ID:52, and TD:58). GI status was determined using the Gastrointestinal Severity Index (GSI), based on the Rome III Diagnostic Questionnaire on Pediatric Functional Gastrointestinal Disorders and other GI symptom questionnaires (26, 27). Parents/guardians completed these questionnaires and were asked to indicate the frequency of GI problems within the last 3 months, including constipation, abdominal pain, vomiting, gaseousness/bloating, and diarrhea on a Likert scale [(0) = never, (1) = rarely, (2) = sometimes, (3) = frequently, and (4) = always)]. The total GSI score was used to evaluate the severity of participants' clinical symptoms. The designation of existing GI problems was used for children rated 3 or higher on more than 1 of the 7 questions.

### Sample Collection and DNA Extraction

Stool samples were collected at Xinhua Hospital of Shanghai Jiao Tong University by their parents of the study participants and kept in 1.8 ml sterile microcentrifuge tubes. After collection, the specimens were immediately frozen at −80°C until further processing. Bacterial DNA was extracted from stool samples using the QIAamp DNA Stool Mini Kit (Qiagen, Hilden, Germany) according to the manufacturers' instructions.

### 16S rRNA Sequencing Analysis

The V3–V4 region of the 16S ribosomal RNA (rRNA) gene was amplified and sequenced on the Illumina MiSeq platform and Ion PGM (Illumina, San Diego, CA, USA). The PE reads obtained first were demultiplexed using the barcodes. After quality filtering, length filtering, dereplication, and removal of model organism sequences, the final sequences were obtained. To determine the identity of bacteria in the remaining high-quality reads, sequences were analyzed using the Quantitative Insights into Microbial Ecology software package (QIIME 2, version 2018.8) and clustered into operational taxonomic units (OTUs) at 97% identity with OTU picking protocol using USEARCH. The taxonomic assignment of the OTUs was carried out using the Naive Bayes classifier (q2-feature-classifier plugin) trained on Greengenes.

### Statistical Analysis

Continuous variables were presented as mean ± standard deviation, and comparisons between ASD, DD/ID, and TD were performed using the Wilcoxon rank-sum test (*p* < 0.05), Mann-Whitney *U*-test or ANOVA. Alpha diversity was analyzed using the core-metrics-phylogenetic for observed diversity metrics, including the evenness diversity metric, chao1 richness estimator, and Shannon's entropy.Weighted and unweighted UniFrac distances of principal coordinate analysis (PCoA) were conducted based on the Bray–Curtis dissimilarity. Between-group differences were investigated using the Kruskal–Wallis test to determine the relative abundance of bacteria at the genus level. Linear discriminant analysis with effect size (LEfSe) was used to determine the difference in microbiota genera with an effect size cut-off of 2.0 and *p*-value of 0.05. Correlations between microbial taxa and clinical parameters were tested using Spearman's test with adjustment for false discovery rate using the Benjamini-Hochberg procedure.

Inferred functional potential of bacterial communities was analyzed using the Phylogenetic Investigation of Communities by Reconstruction of Unobserved States (PICRUSt) with Greengenes database as a reference to obtain a prediction of the Kyoto Encyclopedia of Genes and Genomes (KEGG) ortholog functional profiles (28). Group differences of KEGG pathway were illustrated using the Statistical Analysis of Taxonomic and Functional Profiles (STAMP) package (29).

## Results

### Participant Characteristics

A total of 261 age-matched study participants were enrolled from the Shanghai Xinhua Registry (Table 1), including 138 ASD, 63 DD/ID, and 60 TD children. Overall, the median age of participants was 5.59 years; 191 (73.18%) were male; 111 (51.39%) had natural births, and 105 were delivered by C-section (49.63%). The ASD group was further divided into two groups: ASD with developmental disorder (ASD+DD, *n* = 76) and ASD only (ASD-only, *n* = 62) based on developmental status. As expected, these two subgroups exhibited significant differences in IQ/DQ score (*p* = 2.10e-32) and CARS total score (*p* = 1.28e-8). We also observed differences in the sex distribution of the three groups (*p* < 0.01) due to the sex specificity of ASD.

**Table 1:** Characteristics of ASD, DD/ID, and TD.

**Variables**	**ASD**				**DD/ID** **(*N* = 63)**	**TD** **(*N* = 60)**	***p*-value (ASD vs. DD/ID)**	***p*-value** **(ASD vs. TD)**	***p*-value** **(DD/ID vs. TD)**
	**ASD+DD** **(*N* = 76)**	**ASD-only** **(*N* = 62)**	**Total** **(*N* = 138)**	***p*-value**					
Sex M/F (M %)	63/13 (82.90%)	54/8 (87.10%)	117/21 (84.78%)	n.s.	47/16 (74.60%)	27/33 (45%)	n.s.	2.25E-8	9.48E-4
Age (years)	6.32 ± 2.08	5.86 ± 1.88	6.11 ± 2.00	n.s.	6.39 ± 2.16	6.65 ± 2.22	n.s.	n.s.	n.s.
Delivery mode (Natural/C-section)	33/39	29/29	62/68	n.s.	20/19	29/18	n.s.	n.s.	n.s.
IQ/DQ	50.75 ± 8.92	88.57 ± 14.58	67.74 ± 22.24	2.10E-32	53.53 ± 12.07	105.68 ± 14.87	3.82E-8	5.90E-30	3.33E-41
CARS total	36.16 ± 4.01	32.12 ± 3.75	34.34 ± 4.37	1.28E-8	24.64 ± 2.72	17.14 ± 3.23	1.81E-45	1.63E-66	5.84E-27
Social impairment	23.20 ± 4.75	21.01 ± 2.58	22.24 ± 4.05	1.85E-3	16.86 ± 1.71	11.29 ± 2.83	4.78E-29	5.62E-48	4.73E-25
Negative emotionality	6.72 ± 1.43	6.32 ± 0.99	6.54 ± 1.26	n.s.	4.60 ± 0.72	3.59 ± 1.01	1.74E-30	1.38E-36	8.78E-9
Distorted sensory response	7.01 ± 1.59	6.31 ± 1.08	6.69 ± 1.42	3.70E-3	4.30 ± 1.05	3.21 ± 0.71	4.14E-25	5.20E-56	7.81E-10
GI problem *N*/Total (*N* %)	17/75 (22.67%)	9/62 (14.52%)	26/137 (18.98%)	n.s.	2/52 (3.85%)	4/58 (6.90%)	0.01	0.03	n.s.

*ASD, autism spectrum disorder; DD/ID, developmental delay or intellectual disability; TD, typically developing; CARS, Childhood Autism Rating Scale; ADOS, Autism Diagnostic Observation Scale; GSI, Gastrointestinal Severity Index; n.s., non-significant*.

Scores on the gastrointestinal severity index (GSI) were obtained from 247 children (ASD+DD:75, ASD-only:62, DD/ID:52, and TD:58) (Table 1). Of the 247 participants, 32 (12.96%) were positive for GI symptoms. The children with ASD manifested GI-related problems such as constipation, gaseousness, and diarrhea, whereas the TD children did not show such problems (*p* = 0.013).

### Alterations in Gut Microbial Diversity Between ASD vs. TD and DD/ID Children

**Figure 2:**
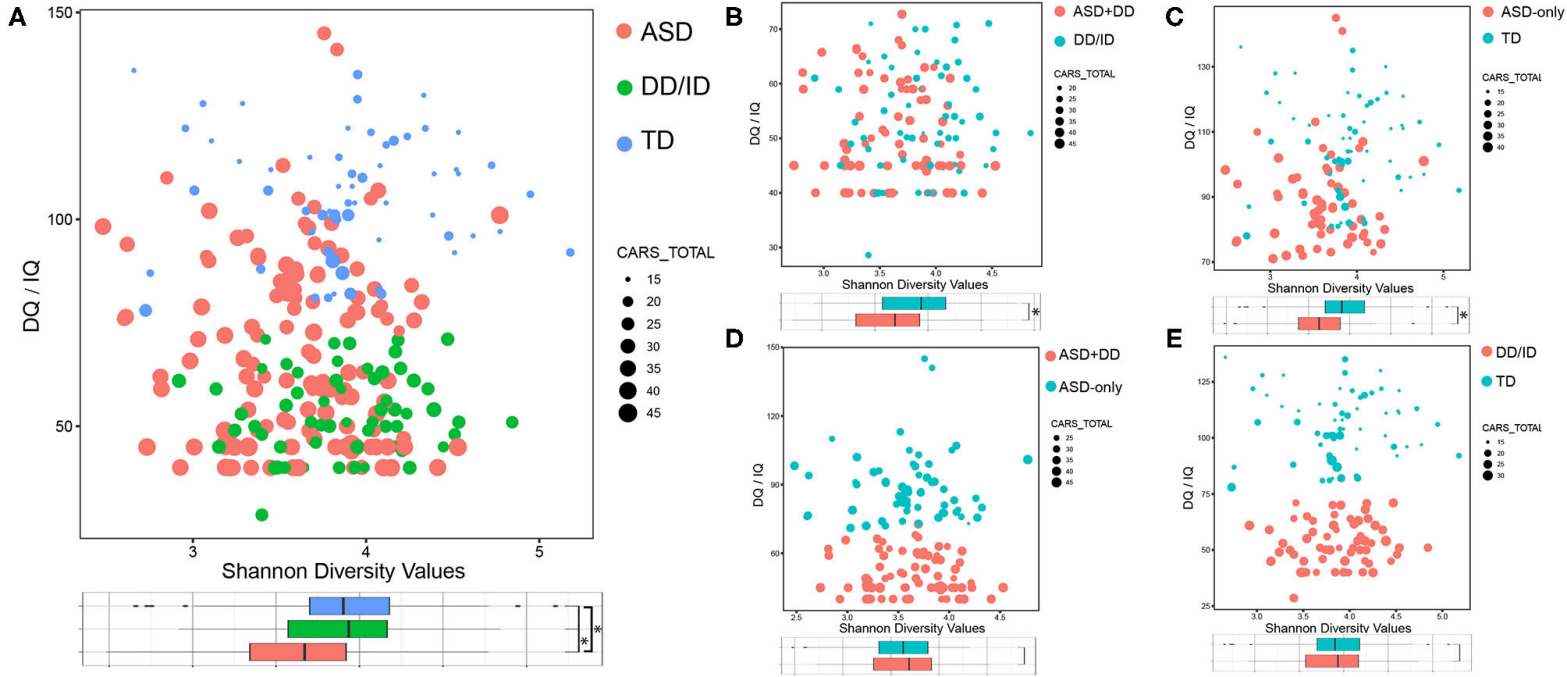
Bacterial richness and diversity comparison according to the Shannon index depicted with IQ/DQ and CARS total scores. The size of node is proportional to the number of CARS total scores. The color of node indicates different groups. *p < 0.05. **(A)** ASD vs. DD/ID vs. TD. **(B)** ASD+DD vs. DD/ID. **(C)** ASD-only vs. TD. **(D)** ASD+DD vs. ASD-only. **(E)** DD/ID vs. TD. ASD, autism spectrum disorder; DD/ID, developmental delay or intellectual disability; TD, typically developing; CARS, Childhood Autism Rating Scale.

To characterize the gut microbiome associated with ASD, we first compared the alpha diversity between children with ASD and the other two groups. Figure 2 shows the distribution of Shannon index, IQ/DQ score, and CARS total score among all three groups of children. We found that the Shannon index for ASD participants decreased compared with those in the DD/ID and TD groups (Figure 2A). Similar results were also observed in five other measures of alpha diversity for the two ASD sub-groups when compared with those in the DD/ID and TD groups, including Chao1, observed species, ACE, Simpson, and InvSimpson indices. All six measures of alpha diversity were consistently decreased in the group of ASD-only children, while the ASD+DD group revealed significant decreases in Shannon, Simpson, and InvSimpson indices (Supplementary Figure 1). The above data indicate that gut microbiome in children with ASD had lower species diversity and evenness. However, no significant difference was detected in the bacterial population or beta diversity analyses between ASD+DD vs. ASD-only or DD/ID vs. TD (Figure 2A and Supplementary Figure 1).

Comparisons were also made between selected subgroups: ASD+DD vs. DD/ID (*p* = 7.50e-4) and ASD-only vs. TD (*p* = 6.60e-4, Figures 2B,C). Both bacterial evenness and alpha diversity indices of the ASD subgroups were significantly lower than the two control groups. These differences suggest that biodiversity might negatively correlate with ASD behaviors, regardless of developmental status. In contrast, there was little difference in any measurement of bacterial diversity within the two ASD sub-groups or within the two control groups (Figure 2D and Supplementary Figure 1), indicating a non-specific action of developmental conditions. Moreover, the PCoA analysis calculated on the Bray–Curtis dissimilarity and unweighted UniFrac distances showed no group difference (Supplementary Figure 2). Overall, our analyses revealed a significantly lower alpha—but not beta—diversity in the gut microbiome of children with ASD compared with DD/ID or TD children.

### Differences in Microbiota Composition Between ASD, TD, and DD/ID Children

We then performed pairwise comparison to investigate the microbial compositions at genus level for all three groups (ASD, DD/ID, and TD). Data are given as median and standard deviation in Table 2. Significantly, in our analysis higher levels of *Bacteroides, Faecalibacterium, Sutterella*, and *Collinsella* were observed in the ASD participants. In contrast, the level of *Prevotella, Coprococcus*, and *Desulfovibrio* were lower than that in the DD/ID or TD groups. The results also revealed an enriched genus *Megamonas* within the DD/ID population.

**Table 2:** Relative abundance of bacteria in fecal microbiota of children with ASD, DD/ID, and TD.

**Variables**	**ASD**	**DD/ID**	**TD**	***p*-value (ASD vs. DD/ID)**	***p*-value (ASD vs. TD)**	***p*-value (DD/ID vs. TD)**
*Bacteroides*	0.381 ± 0.166	0.306 ± 0.173	0.311 ± 0.182	0.0090	0.0153	n.s.
*Faecalibacterium*	0.080 ± 0.071	0.044 ± 0.040	0.059 ± 0.052	0.0001	0.0364	n.s.
*Sutterella*	0.032 ± 0.029	0.022 ± 0.022	0.030 ± 0.025	0.0204	n.s.	0.0206
*Prevotella*	0.033 ± 0.102	0.053 ± 0.122	0.055 ± 0.139	0.0043	0.0036	n.s.
*Megamonas*	0.014 ± 0.042	0.025 ± 0.052	0.009 ± 0.036	0.0015	n.s.	0.0178
*Coprococcus*	0.006 ± 0.014	0.010 ± 0.012	0.011 ± 0.013	0.0181	0.0049	n.s.
*Collinsella*	0.007 ± 0.030	0.007 ± 0.010	0.007 ± 0.011	0.0080	0.0246	n.s.
*Desulfovibrio*	0.005 ± 0.009	0.006 ± 0.008	0.007 ± 0.007	0.0069	0.0046	n.s.

*Data show only the significantly (P < 0.05) different abundances at genus level. P-values were obtained from the Mann–Whitney U-test. ASD, autism spectrum disorder; DD/ID, developmental delay or intellectual disability; TD, typically developing; n.s., non-significant*.

**Figure 3:**
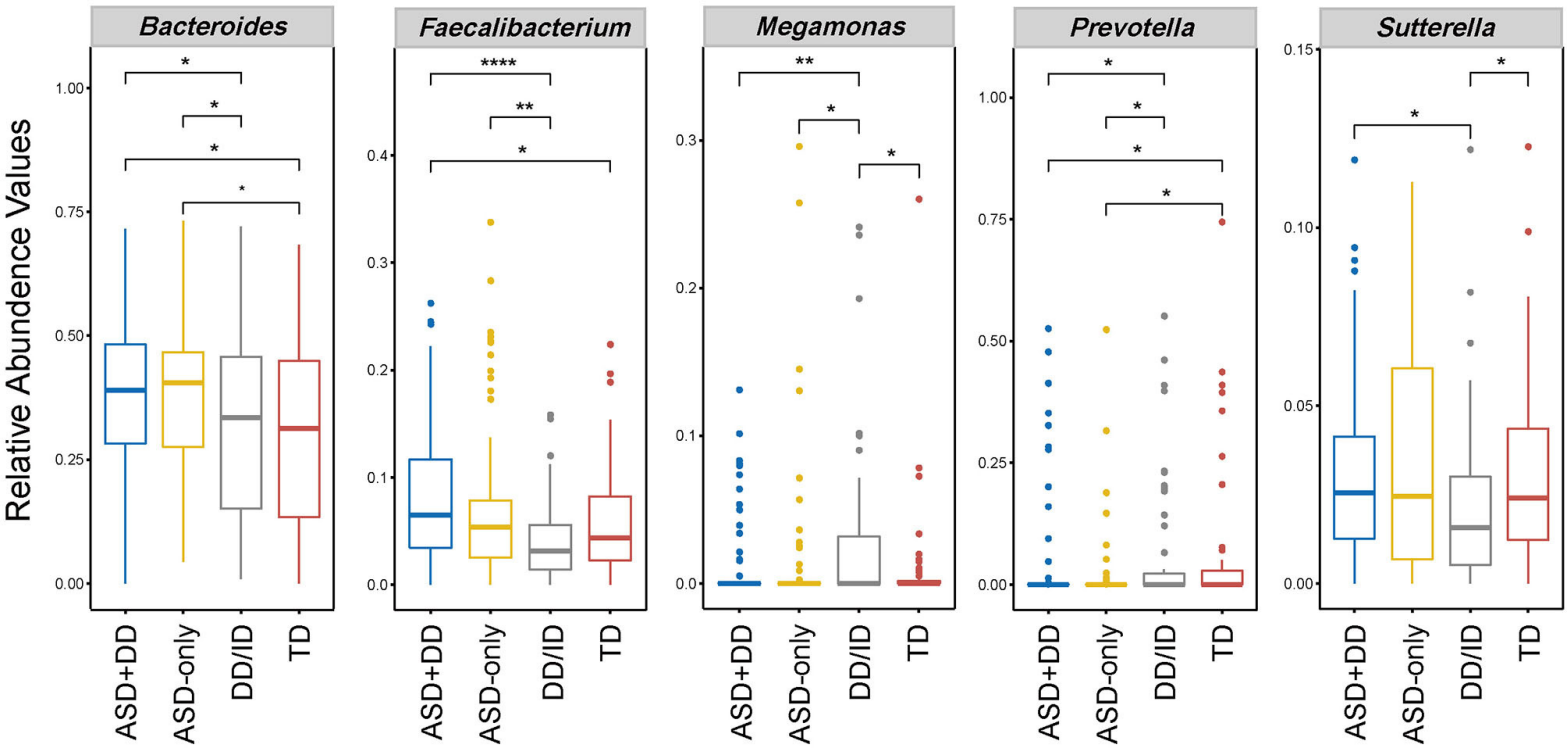
Box plot of selected bacteria according to relative abundances of genus taxon in feces of ASD subgroups, DD/ID, and TD. *p < 0.05, **p < 0.01, and ****p < 0.0001. This figure shows only genus abundances with a value higher than 0.1%.

Next, LEfSe analysis was used to determine significant alterations in the bacterial composition associated with ASD sub-groups, DD/ID and TD groups (Supplementary Figure 3). Considering the differences in relative abundance, five genera, including *Bacteroides, Faecalibacterium, Prevotella, Sutterella*, and *Megamonas* are shown in Figure 3. The abundance of *Bacteroides* and *Prevotella* ensured differences between the two ASD and non-ASD groups. An increased abundance of genus *Sutterella* was observed in ASD samples, and yet the comparison only approached significance in the context of developmental delay (ASD+DD vs. DD/ID). The abundance of *Faecalibacterium* was significantly increased in the ASD participants compared with non-ASD participants (Table 2). Still, no statistically significant difference was found in the context of typical development (ASD-only vs. TD). In addition, the levels of *Sutterella* and *Megamonas* varied markedly when comparison was made between the DD/ID and TD groups. However, there was no significant difference in microbiota composition between ASD sub-groups after FDR correction. Taken together, the above results confirm a microbial dysbiosis in children with ASD and, to a lesser extent, in the ASD subgroup in comparison with the non-ASD groups.

### Correlations Between Gut Microbiota Composition and Clinical Profiles

**Figure 4:**
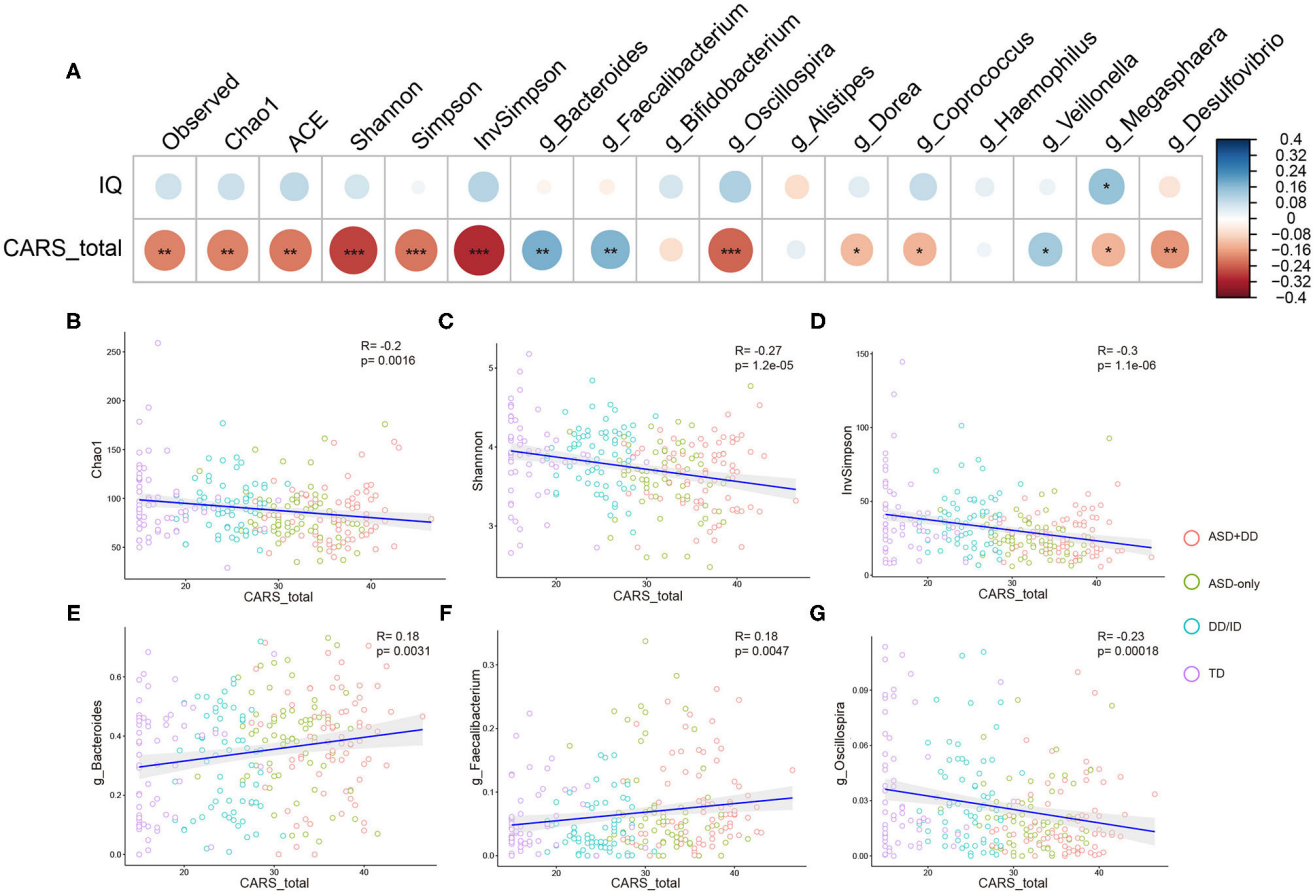
Correlation between alpha diversity, microbiota genera, and clinical parameters in all participants. Heatmaps showing association between alpha diversity, microbiota composition, and clinical characteristics **(A)**. *p < 0.05 and **p < 0.01. Total CARS score is negatively correlated with Chao1 index **(B)**, Shannon index **(C)**, InvSimpson index **(D)** in all participants. Genus *Bacteroides* **(E)**, genus *Faecalibacterium* **(F)**, and genus *Oscillospira* **(G)** based on the OTU profile were associated with CARS total scores. *p < 0.05, **p < 0.01, and ***p < 0.001.

Spearman (dichotomous variables) or Pearson (continuous variables) correlation analyses were used to explore the relationship between the gut microbiota composition and phylogenetic diversity at genus level, and the clinical profiles in ASD, DD/ID, and TD children. As the CARS total score represents the severity of ASD symptoms and the IQ score indicates intelligence, correlations of microbial profiles, CARS total score, and IQ were investigated for all participants. However, DQ and IQ were calculated using different scales, 104 ASD (ASD+DD:66, ASD-only:38), 53 DD/ID, and 56 TD participants whose IQs had been obtained were included. Indicators of bacterial biodiversity were found to strongly and significantly correlate with the CARS total score (Figures 4A–D). Similar correlations with CARS total score were also observed with selected genera, including *Bacteroides, Faecalibacterium*, and *Oscillospira* (Figures 4A,E,F), suggesting a broader involvement of those microbiota in ASD symptoms.

**Figure 5:**
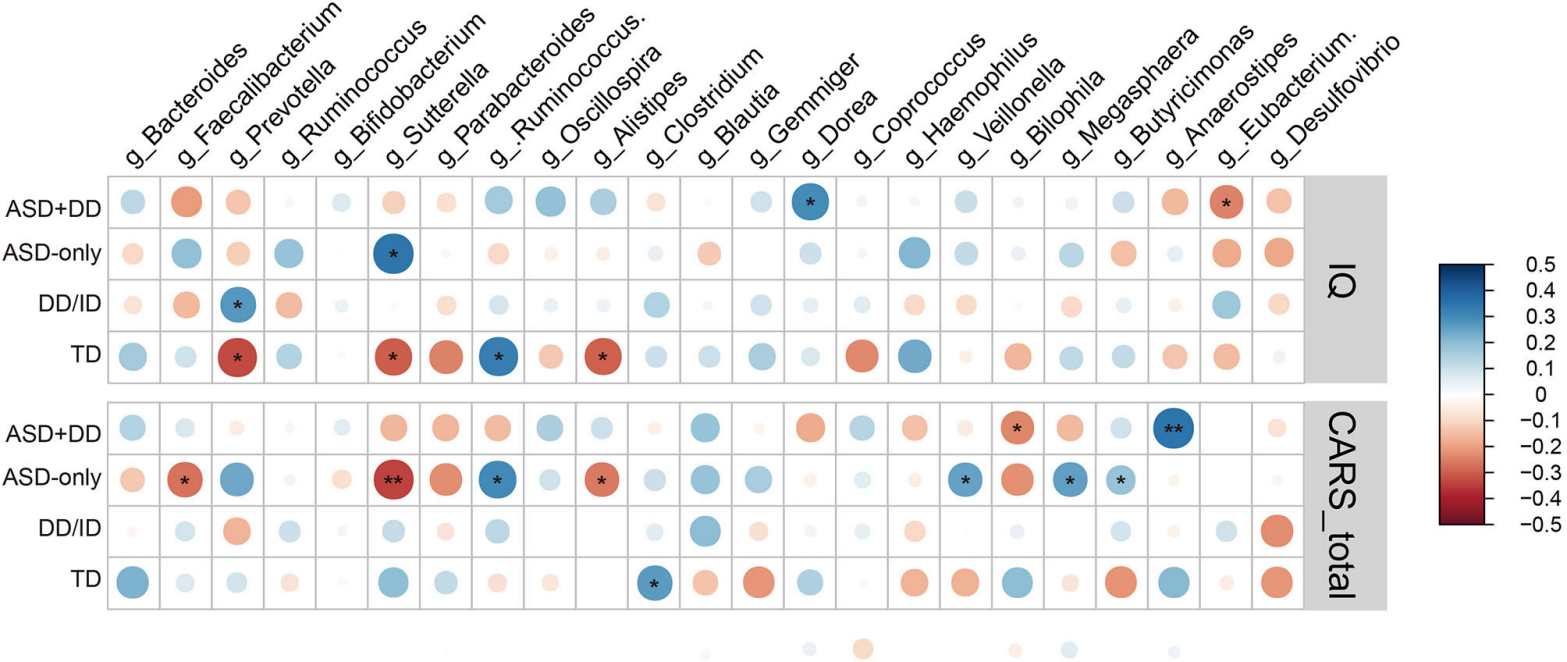
Heatmaps showing correlations between microbiota genus and clinical characteristics in group ASD+DD, ASD-only, DD/ID, and TD. *p < 0.05 and **p < 0.01.

Next, we calculated potential relationships in relative abundance based on OTU counts of the microbiota at genus level and the clinical profiles across participants in each individual group. There was a significant association between the CARS total score and the abundance of *Sutterella* in the group of ASD children (*r* = −0.24, *p* = 0.0046). The result remained consistent when analyses were stratified by intellectual status, especially in the ASD-only group (*r* = −0.34, *p* = 0.0072). In addition, genus *Anaerostipes* was positively correlated with the CARS total scores in the ASD+DD group (*r* = 0.35, *p* = 0.0018, Figure 5).

Moreover, the correlations between microbial profile and IQ score of participants were analyzed. The abundance of *Prevotella* was positively correlated with IQ score in the DD/ID group (*r* = 0.27, *p* = 0.048) and negatively correlated (*r* = −0.33, *p* = 0.014) in the TD group. The higher abundance of *Sutterella* was associated with higher IQ scores in the ASD-only group (*r* = 0.346, *p* = 0.033) but related to lower intellectual state in the TD group (*r* = −0.297, *p* = 0.026, Figure 5). Taken together, these observations imply an impact of these microbiota on social ability and intellectual state, for the ASD population and/or those with intellectual disability.

### Predictive Microbiota Functional Profiling

The functional contributions of the bacteria in the ASD, DD/ID, and TD children were predicted based on OTUs using the KEGG microbial database. The KEGG pathways were filtered to include those with five or more alignments in at least 10% of the samples and 330 KEGG pathways were found across all samples. The effect sizes identified as significantly different (*p* < 0.05) between groups were assessed to determine discriminatory KEGG orthology.

**Figure 6:**
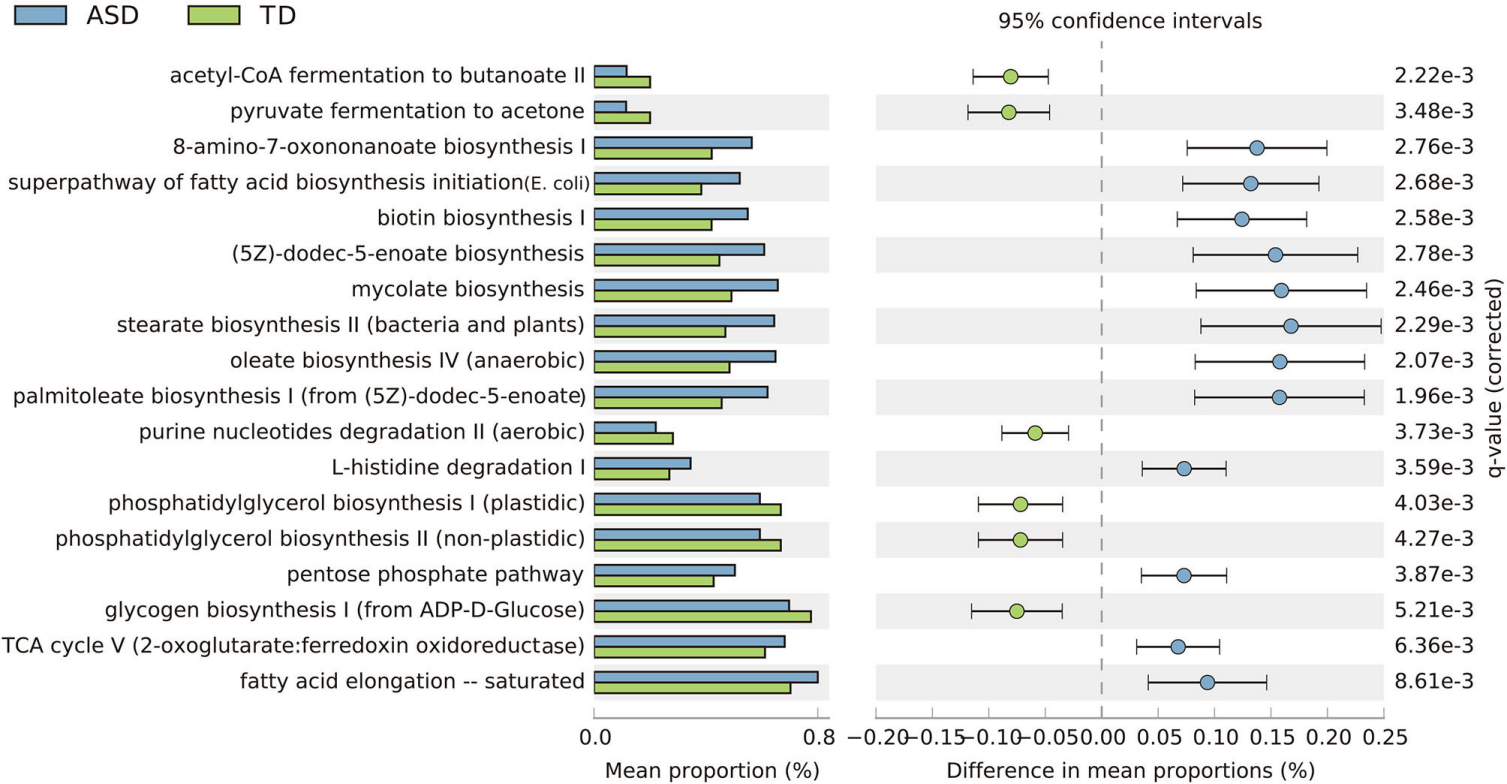
Extended error bar plot showing 18 significantly different KEGG categories and abundance of the expression in the ASD and TD groups (*p* < 0.001, effect size >0.05. Benjamini–Hochberg FDR <0.05).

Based on the threshold value of FDR <0.05, 18 key KEGG pathways demonstrated differential abundance between the ASD and TD groups, mainly belonging to organismal systems, cellular processes, and metabolism. KEGG pathways, including fatty acid biosynthesis, biotin biosynthesis, and L-histidine degradation, were significantly enriched in children with ASD. In contrast, several pathways—including glycogen biosynthesis, phosphatidylglycerol biosynthesis, and Nucleotide Metabolism—were relatively reduced in children with ASD and significantly enriched in TD children (Figure 6).

Next, the differences in representation among the microbiome in children with ASD and DD/ID were interrogated. KEGG pathways were found to vary with the same trend (Supplementary Figure 4A). The differences in representation between the groups of DD/ID vs. TD and groups of ASD+DD vs. ASD-only are displayed in Supplementary Figures 4B,C, respectively. However, no statistical significance was identified between these two pairwise comparisons after FDR correction.

## Discussion

Although the alterations in gut microbiota of human and animal models of ASD relative to non-ASD participants have been explored in the past decade (22, 30), human studies focusing on how particular microbial pattern links to specific ASD symptom or comorbidity remain largely lacking. In the present study, we performed bacterial taxonomy assignments using the stool samples from two age-matched control groups of DD/ID and TD participants. Associations between the microbial profile and the severity of social impairment, intellectual disability, and GI symptoms in children with ASD were systemically assessed.

The rapidly expanding body of literature on basic, translational, and clinical research on the gut microbiome suggests that the microbiota could influence brain development and have a significant impact on neurological and psychiatric diseases (31, 32). For instance, Vogt et al. characterized the bacterial taxonomic composition of stool samples from patients with Alzheimer's disease (AD). They revealed that the AD gut microbiome had decreased microbial diversity and was compositionally distinct from age- and sex-matched control individuals (33). Further analyses identified genus-wide differences in bacterial abundance, including decreased *Firmicutes* and *Bifidobacterium*, and increased *Bacteroidetes* in the gut microbiome of AD participants (34). In a longitudinal study, Carlson et al. demonstrated that the gut microbial composition of the human infant at 1 year of age could predict cognitive performance at age 2 (35). A more recent study identified a 12-genus microbial signature associated with brain structure variations and behavioral characteristics using a machine learning framework in children with ASD (36). We thus hypothesize that specific microbial pattern or signature taxa may contribute to specific dimension of ASD and other comorbidities.

The present study was a pilot exploration where we first aimed to determine the differential role of gut microbiota in social impairment and intellectual disability, as DD/ID has emerged as the most common comorbidity of ASDs. Following previous reports (37), our analysis showed a significantly lower abundance of genus *Prevotella* in children with ASD (Figure 4). The abundance of *Prevotella* correlated positively with the IQ score in DD/ID participants (*p* = 0.048) but correlated inversely in TD participants (*p* = 0.014). Similarly, *Prevotella* abundance has been shown to positively correlate with fluid cognition scores in chronic marijuana users but not in non-users (38). *Sutterella* abundance was also reported to have a negative association with MoCA score in patients with Sporadic Parkinson's Disease (39). Interestingly, we observed a similar negative correlation of *Sutterella* abundance and IQ score in TD children (*p* = 0.026). However, this correlation was not detected or even reversed in other subgroups of the study participants.

The cladograms generated by LEfSe indicated differences in the bacterial taxa between TD vs. DD/ID participants and ASD-only vs. ASD+DD participants (Supplementary Figure 3). Although it is generally believed that the genetic causes are varied and similar for DD/ID and ASDs (4), these two comparisons revealed distinct signature taxa and a unique functional enrichment of the metabolic pathways associated with DD/ID (Supplementary Figures 3, 4), suggesting they may have evolved through different mechanisms of gut microbiota in DD/ID vs. in ASD with comorbid DD/ID.

We compared the alpha and beta diversities of ASD participants in relation to control participants to characterize the overall alterations in the microbiota of children with ASD and to determine the associations between the gut microbial profile and severity of social impairment in ASD. In metagenomics, alpha diversity represents the richness and diversity of the microbiome at the smallest spatial scale of analysis, in which Chaos1, ACE, Simpson, and Shannon indices are commonly used to calculate the alpha diversity (40). Previous studies lacked consistency when comparing children with ASD vs. healthy controls and some have described opposing results. For instance, several early 16S rRNA amplicon studies showed that the gut microbiome of children with ASD exhibited increases of richness (ACE and Chao1 indices), evenness (Shannon even index), and diversity (Shannon index) (20, 41) or no significant changes of Shannon index and observed species (34, 42, 43). However, relatively low microbial richness (ACE estimator) and diversity (PD and Shannon indices) have been reported in children with ASD in recent investigations (44–46). A possible reason for these inconsistent results may be due to the lack of homogeneity in terms of age, geographic area, and severity of behavioral symptoms among the ASD participants, and differences in control group selection. Altered bacterial diversity have also been described in deep metagenomic sequencing study using stool samples from ASD children, which revealed increased bacterial richness presented by Chao1 (47) and inverse Simpson diversity indices (48). Nevertheless, previous studies have also reported that the biodiversity (fisher's alpha) (49) and species richness (Chao1) (50) were significantly lower in ASD patients, suggesting different research methodologies could yield contradictory results.

In the present study, analyses of alpha diversity (Chao1, observed species, ACE, Shannon, Simpson, and InvSimpson indices) showed a minor but statistically significant decrease in the bacterial richness of children with ASD compared with non-ASD participants (Figure 2 and Supplementary Figure 1). As shown in Supplementary Figure 5, we further examined the associations of the alpha diversities (Chao1, observed species, ACE, Shannon, Simpson, and InvSimpson indices) with the CARS total scores in participants with DD/ID (the combination of DD/ID and ASD+DD children), and then in participants without DD/ID (the combination of TD and ASD-only children). These results indicated that the association of Shannon diversity index of microbiome and the severity of social impairment is independent of IQ in children with ASD. The beta diversity refers to the total variance in the microbial community composition across varying environments. Several papers showed significant differences in beta diversity between ASD and control participants—although some did not (42, 51). Our analyses revealed no significant difference (Supplementary Figure 2). Correlations of microbiota were also calculated with the CARS total scores and the IQ scores. In the groups of DD/ID and ASD+DD children, genus *Bacteroides* (*r* = 0.22, *p* = 0.011) and genus *Faecalibacterium* (*r* = 0.32, *p* = 9.6e-5) were found positively associated with the CARS total scores. However, in combination of TD and ASD-only participants, genus *Streptococcus* (*r* = 0.24, *p* = 0.022) was positively correlated with the IQ scores.

Having shown that the microbiome was significantly correlated with the CARS total score in children with ASD, we further explored the associations of bacterial taxa and behavioral measurements in the three primary CARS domains representing diverse behavioral and emotional aspects in two subgroups of children with ASD (ASD+DD and ASD-only). At genus level, the abundance of *Faecalibacterium* in children with ASD only correlated negatively with the domain of Social impairment (*r* = −0.33, *p* = 0.0092). Still, it was not associated with the other two CARS domains (Negative emotionality and Distorted sensory responses). In contrast, *Sutterella* and *Prevotella* were significantly associated with all three domains in CARS (Supplementary Figure 6). However, these correlations were not identified in children of the ASD+DD subgroup. These analyses suggest unique and diverse correlations between the microbial pattern and behavioral and emotional features of ASD, while further investigation and validation are warranted.

Preliminary studies indicate that children with ASD frequently have GI problems and gut microbiota dysbiosis (52, 53). However, it has been reported that the microbial changes were more closely linked to the presence of ASD symptoms rather than to the severity of GI symptoms (54). Our analyses identified the genera *Bifidobacterium* (*r* = 0.26, *p* = 0.0023) and *Alistipes* (*r* = 0.28, *p* = 0.00085) had a strong and positive correlation with the severity of GI symptoms evaluated by the GSI total score of the ASD group, allowing us to speculate about the possible contributions of *Bifidobacterium* and *Alistipes* to GI symptoms. However, our current study was an initial attempt to discern the correlations in microbial changes with GI symptoms and other clinical manifestations in children with ASD. Due to limited validation of the GSI, future studies require more effective and valid questionnaires to ensure precise information.

There are some limitations to the present study. First, 16S rRNA gene amplicon sequencing has limited classification power and cannot fully resolve taxonomic profiles at the species or strain level depending on the database and classifiers used (55, 56). Thus, shotgun sequencing remains needed for comprehensive profiling of gut microbiota and to unveil the subtle differences between each subpopulation. Second, children with ASD are not a homogenous clinical population, but a group of individuals with similar behavioral characteristics and a potentially broad range of pathologies. Although associations of genera with IQ performance were identified in specific subgroups of ASD, the results warrant further validation in other independent cohort studies with larger sample sizes and stratification.

Overall, we observed alterations in bacterial taxonomic composition in children with ASD, and their associations with specific clinical manifestations. These results illustrate that gut microbiota could serve as a promising biomarker and possible therapeutic target for ASD symptoms.

## Data Availability Statement

The datasets presented in this study can be found in online repositories. The names of the repository/repositories and accession number(s) can be found at: NCBI BioProject, PRJNA769228.

## Ethics Statement

The studies involving human participants were reviewed and approved by Ethical Committee of Shanghai Jiao Tong University School of Medicine Affiliated Xinhua Hospital. Written informed consent to participate in this study was provided by the participants' legal guardian/next of kin.

## Author Contributions

ZC and JY designed the study. ZC, XL, YD, YL, LZ, and XD collected the fecal samples and clinical information. KS, JY, and ZC carried out the analysis, interpreted the results, and wrote the manuscript. TZ and SF analyzed the data and helped design experiments. FL and JY guided and supervised all work. All authors contributed to the article and approved the submitted version.

## Funding

This study was supported by grants from the National Natural Science Foundation of China (82125032, 81930095, 31860306, 81761128035, and 82001771), the Science and Technology Commission of Shanghai Municipality (19410713500 and 2018SHZDZX01), the Shanghai Municipal Commission of Health and Family Planning (GWV-10.1-XK07, 2020CXJQ01, 2018YJRC03, and 2018BR33), the Guangdong Key Project (2018B030335001), Science and Technology Department of Yunnan Province (202001AV070010), and Danone Institute China Diet Nutrition Research and Communication Grant DIC2017-10.

## Conflict of Interest

The authors declare that the research was conducted in the absence of any commercial or financial relationships that could be construed as a potential conflict of interest. The reviewer MW declared a shared affiliation, with no collaboration, with the author KS at the time of the review.

## Publisher's Note

All claims expressed in this article are solely those of the authors and do not necessarily represent those of their affiliated organizations, or those of the publisher, the editors and the reviewers. Any product that may be evaluated in this article, or claim that may be made by its manufacturer, is not guaranteed or endorsed by the publisher.

## Supplementary Material

Supplementary Figure 1 Six measures of alpha diversity: Observed species index, Chao1 index, ACE index, Shannon index, Simpson index, and InvSimpson index in group ASD+DD, ASD-only, TD, and DD/ID. Each box plot represents the median, interquartile range, minimum, and maximum values. ^*^*p* < 0.05.

Supplementary Figure 2 Beta diversity analysis on fecal samples from ASD subgroups, DD/ID, and TD children. Bray-Curtis β-diversity indices of the fecal microbiota showed no distinction between four groups. ASD, autism spectrum disorder; DD/ID, developmental delay or intellectual disability; TD, typically developing.

Supplementary Figure 3 Pairwise comparison by LEfSe analysis between groups with autism spectrum disorder (ASD), developmental delay or intellectual disability (DD/ID), and typically developing (TD) children (score >2.0 and *p* < 0.05). The cladogram of enriched taxa between ASD+DD and ASD-only **(A)**, DD/ID and TD **(B)**, ASD+DD and DD/ID **(C)**, ASD-only and TD **(D)** gut microbiome.

Supplementary Figure 4 Extended error bar plot representing the different KEGG category abundance. **(A)** There were 42 different KEGG pathways identified in the ASD and DD/ID groups (*P* < 0.001, effect size >0.05. Benjamini–Hochberg FDR <0.05). **(B)** 9 KEGG pathways for differences among children with DD/ID and TD (*p* < 0.05). **(C)** 10 KEGG pathways were distinct within ASD subgroups (*p* < 0.05).

Supplementary Figure 5 Correlation between the richness and diversity of bacterial and clinical characteristics in selected groups. **(A)** Correlation in participants with comorbid DD/ID (DD/ID and ASD+DD). **(B)** Correlation in participants without comorbid DD/ID (TD and ASD-only). Shannon index was negatively correlated with the total CARS score in group whether combined DD/ID **(C)** or not **(D)**. Total CARS score was negatively correlated with Simpson index in group whether combined DD/ID **(E)** or not **(F)**.

Supplementary Figure 6 Association between bacterial relative abundances and three primary CARS domains in subgroups of children with ASD. **(A)** Heatmaps showing correlations between genus and Social impairment, Negative emotionality, and Distorted sensory responses. In the ASD-only group, the abundance of *Faecalibacterium* was negatively correlated with social impairment **(B)**, negative emotionality **(C)**, and distorted sensory responses **(D)** in the ASD-only group. The *Sutterella* genus showed significant negative correlations with social impairment **(E)**, negative emotionality **(F)**, and distorted sensory responses **(G)**.
