# Supplementary figures and images for: Gut Microbial Profile Is Associated With the Severity of Social Impairment and IQ Performance in Children With Autism Spectrum Disorder
Source: Front Psychiatry. 2021 Dec 17;12:789864. doi: 10.3389/fpsyt.2021.789864 (PMC8718873; doi:10.3389/fpsyt.2021.789864)

Alpha Diversity Values

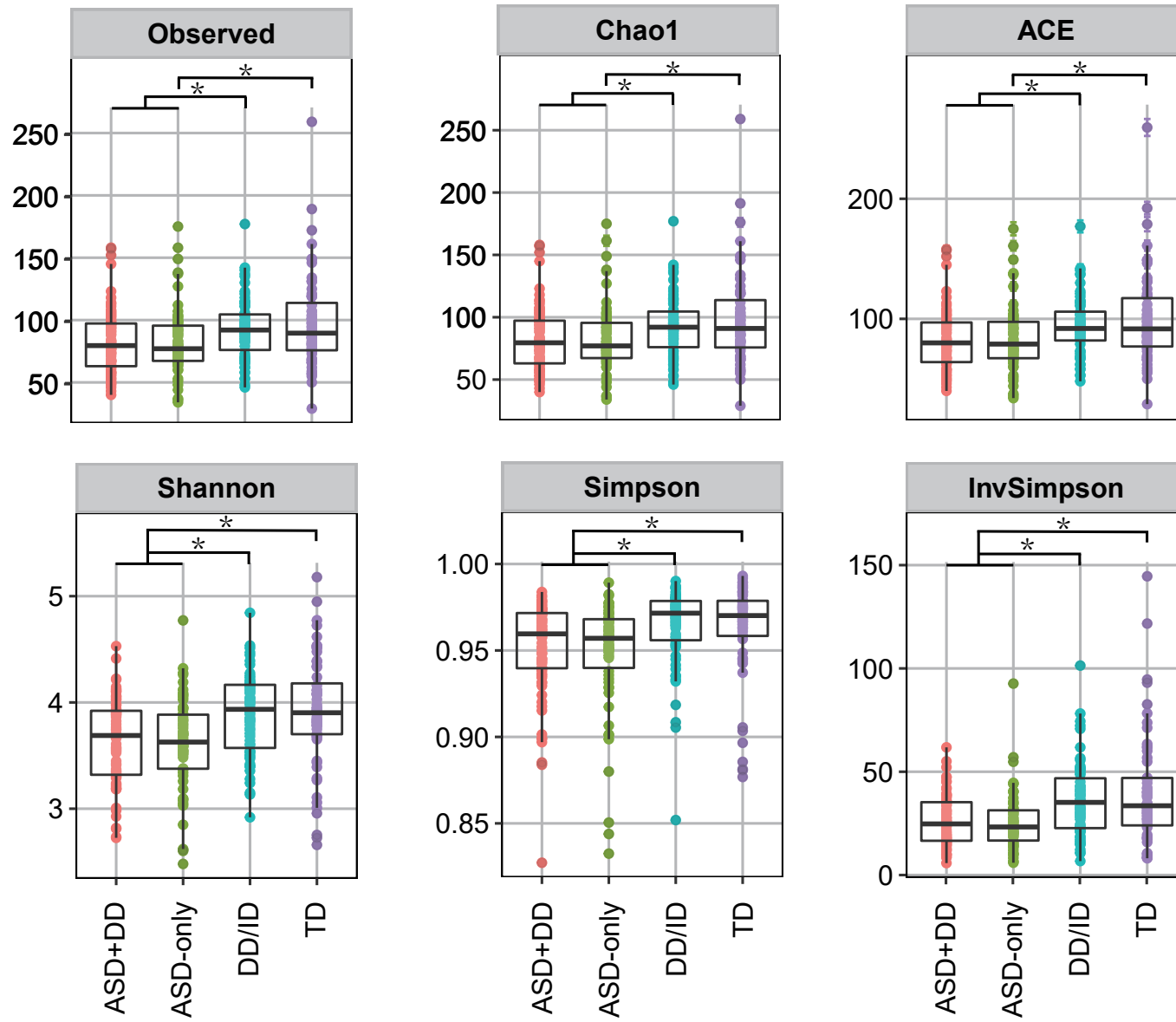

Supplement: Supplementary Figure 1 — Six measures of alpha diversity: Observed species index, Chao1 index, ACE index, Shannon index, Simpson index, and InvSimpson index in group ASD+DD, ASD-only, TD, and DD/ID. Each box plot represents the median, interquartile range, minimum, and maximum values. *p < 0.05. [file Data_Sheet_1.PDF]

**A**

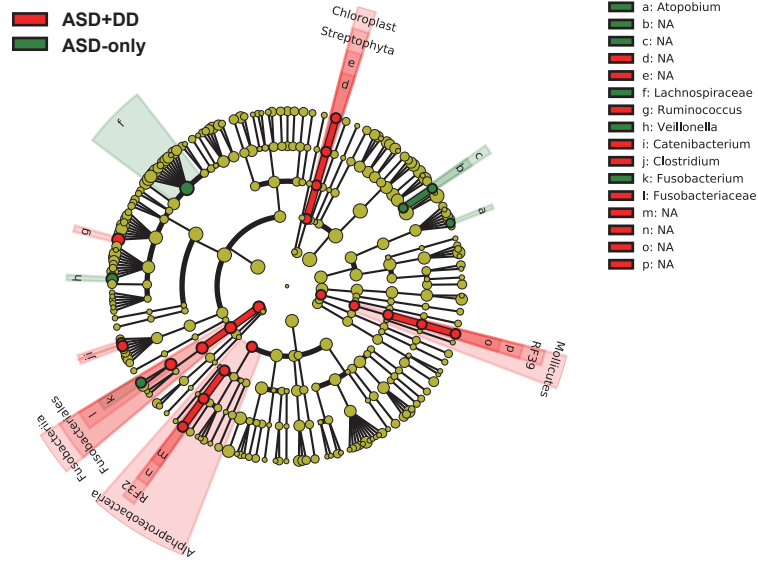

**B**

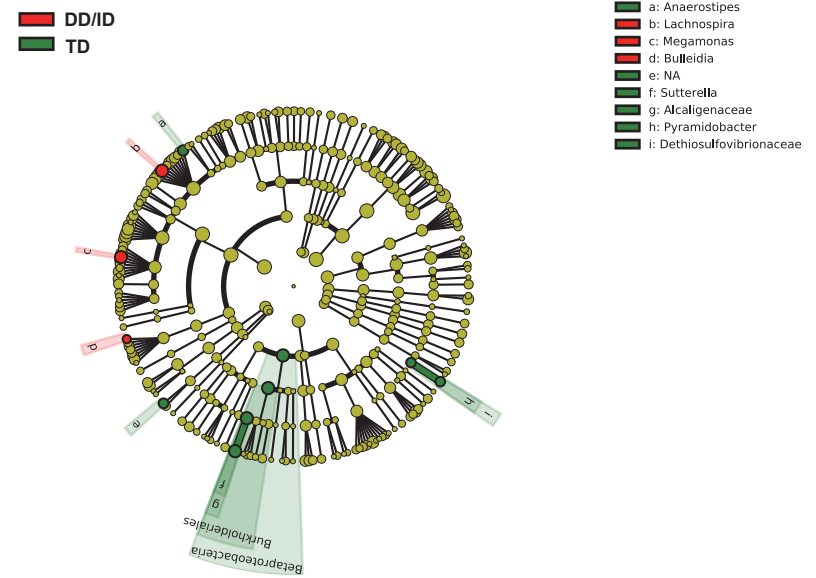

C

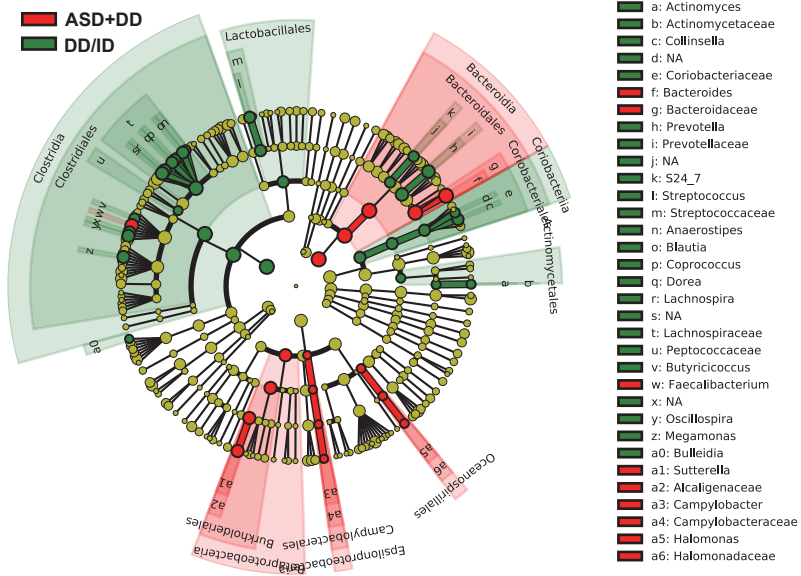

D

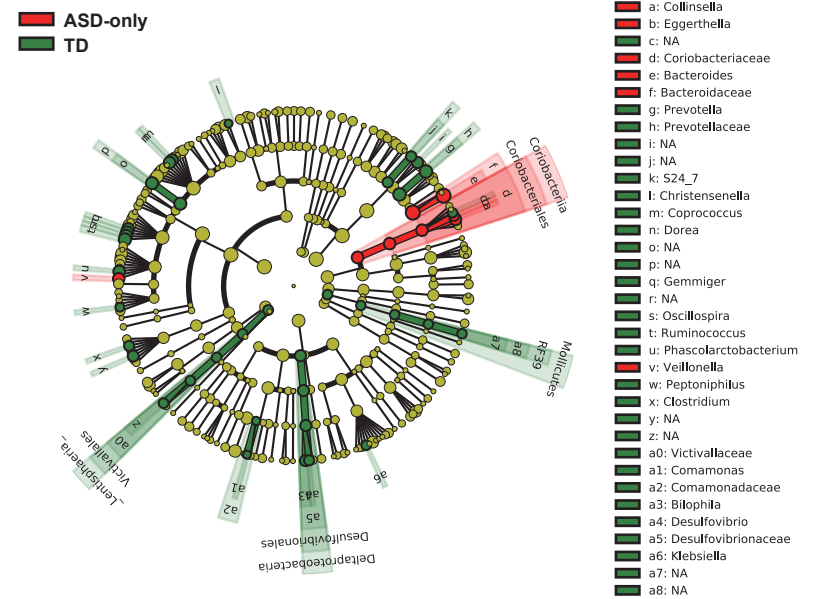

Supplement: Supplementary Figure 2 — Beta diversity analysis on fecal samples from ASD subgroups, DD/ID, and TD children. Bray-Curtis β-diversity indices of the fecal microbiota showed no distinction between four groups. ASD, autism spectrum disorder; DD/ID, developmental delay or intellectual disability; TD, typically developing. [file Data_Sheet_2.PDF]

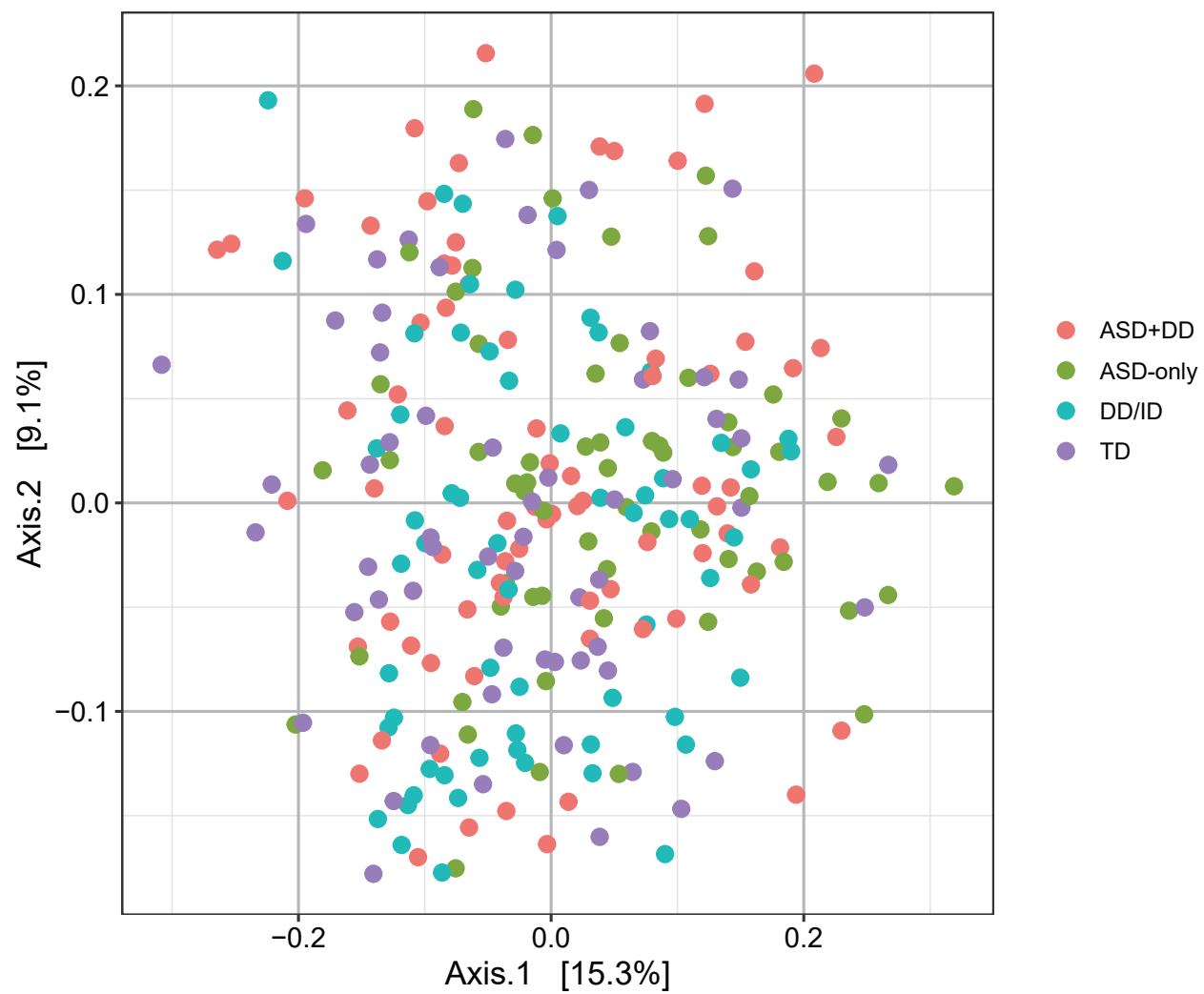

Supplement: Supplementary Figure 3 — Pairwise comparison by LEfSe analysis between groups with autism spectrum disorder (ASD), developmental delay or intellectual disability (DD/ID), and typically developing (TD) children (score >2.0 and p < 0.05). The cladogram of enriched taxa between ASD+DD and ASD-only (A), DD/ID and TD (B), ASD+DD and DD/ID (C), ASD-only and TD (D) gut microbiome. [file Data_Sheet_3.PDF]

A

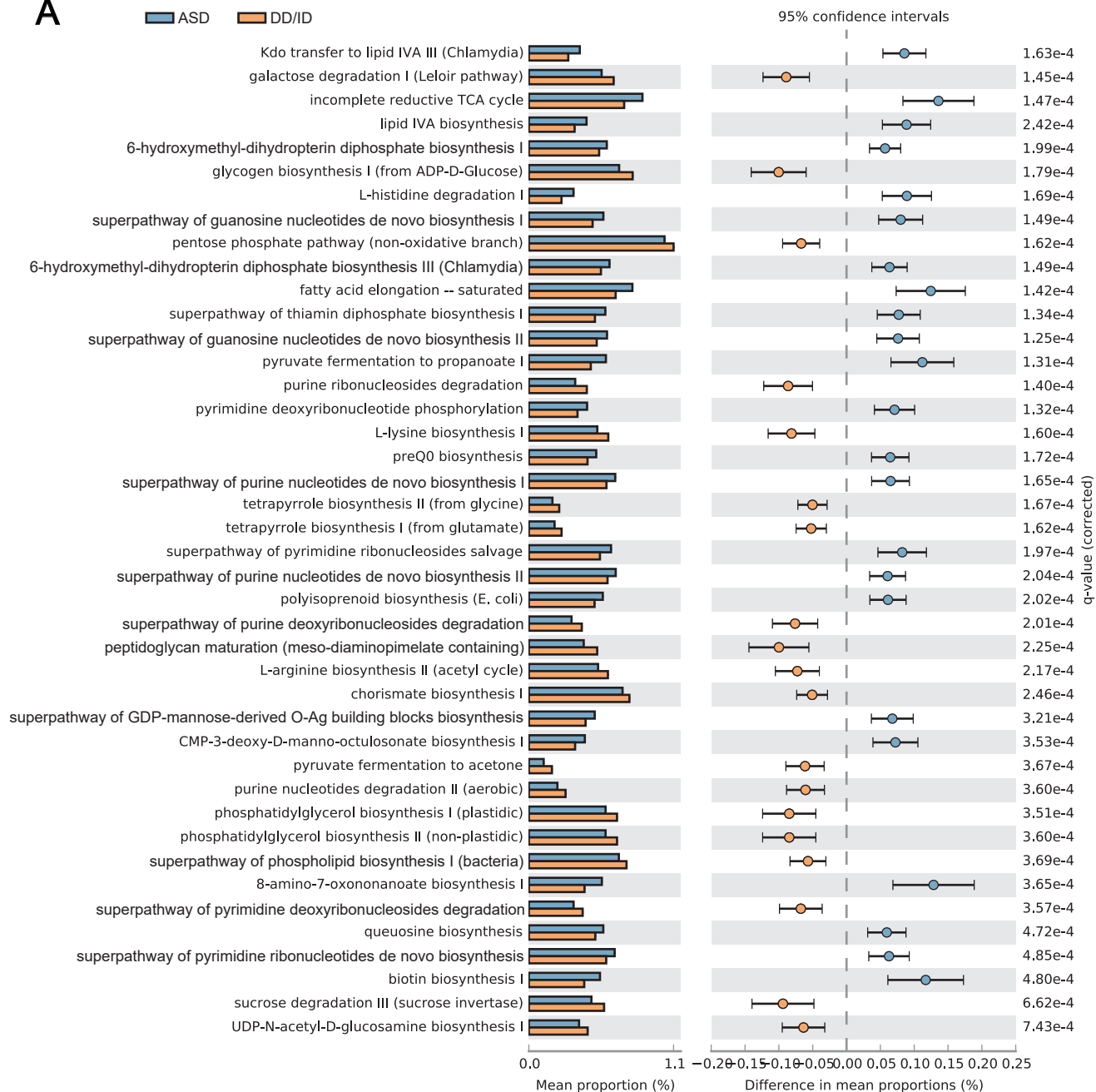

B

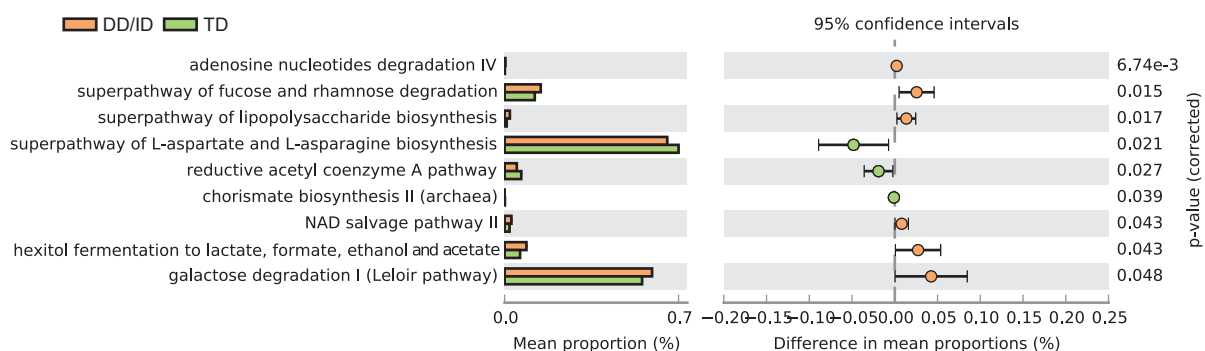

C

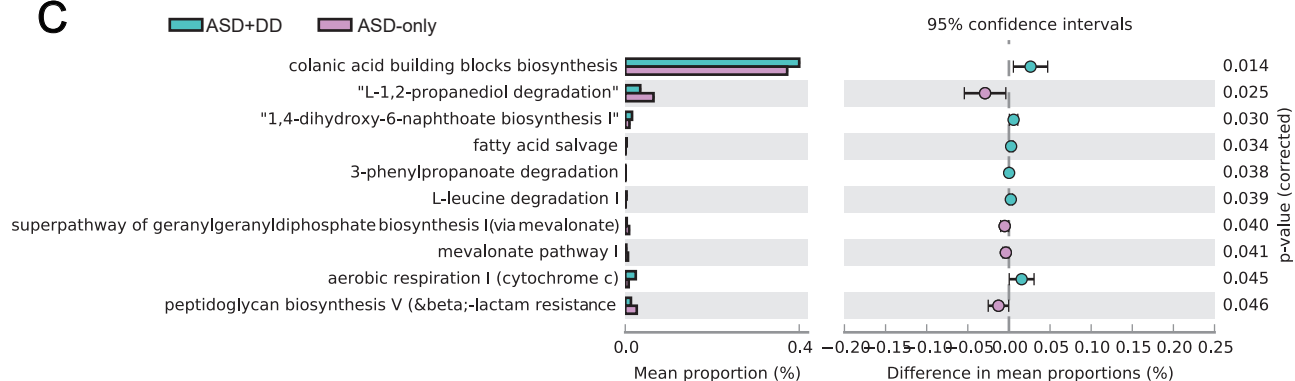

Supplement: Supplementary Figure 4 — Extended error bar plot representing the different KEGG category abundance. (A) There were 42 different KEGG pathways identified in the ASD and DD/ID groups (P < 0.001, effect size >0.05. Benjamini–Hochberg FDR <0.05). (B) 9 KEGG pathways for differences among children with DD/ID and TD (p < 0.05). (C) 10 KEGG pathways were distinct within ASD subgroups (p < 0.05). [file Data_Sheet_4.PDF]

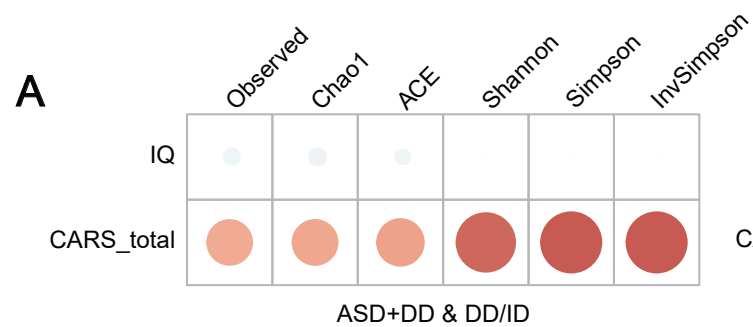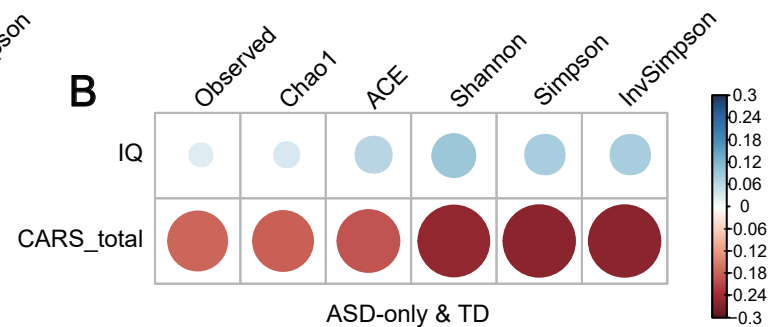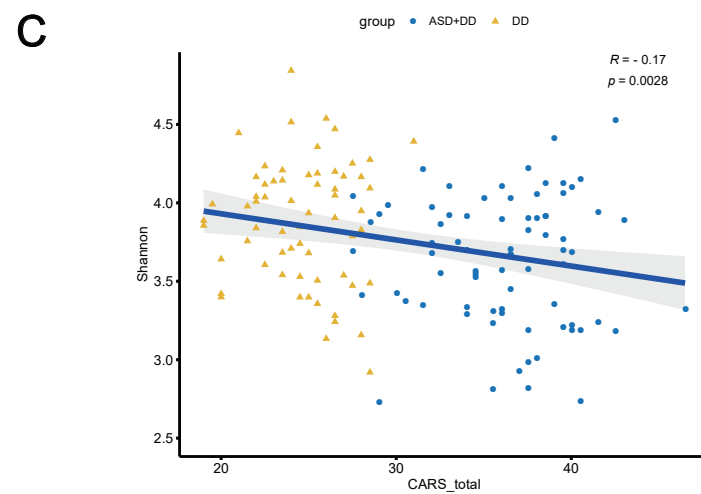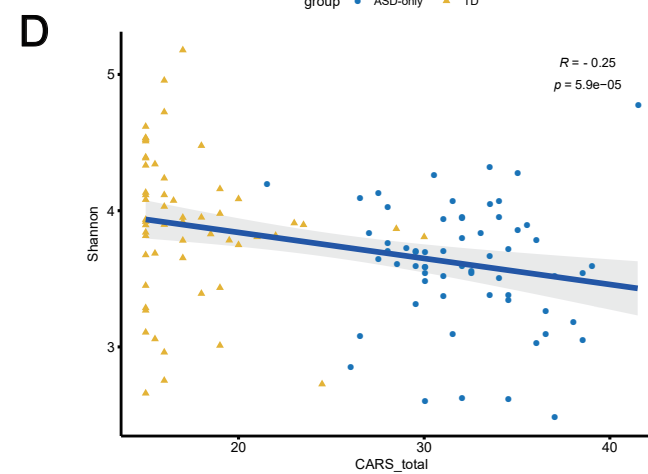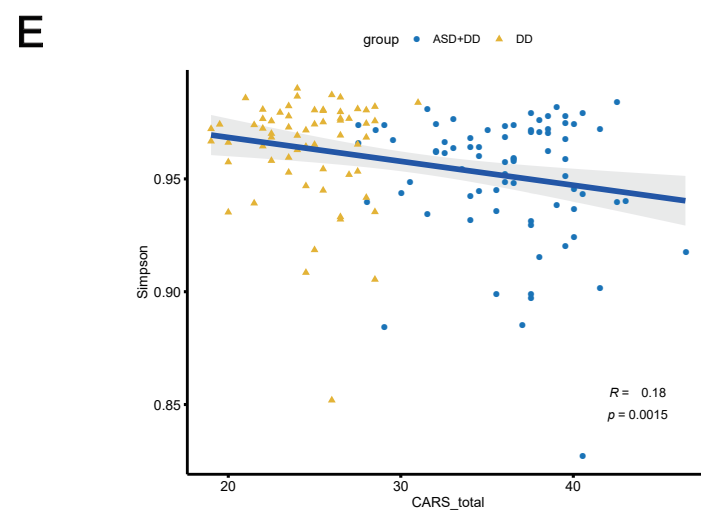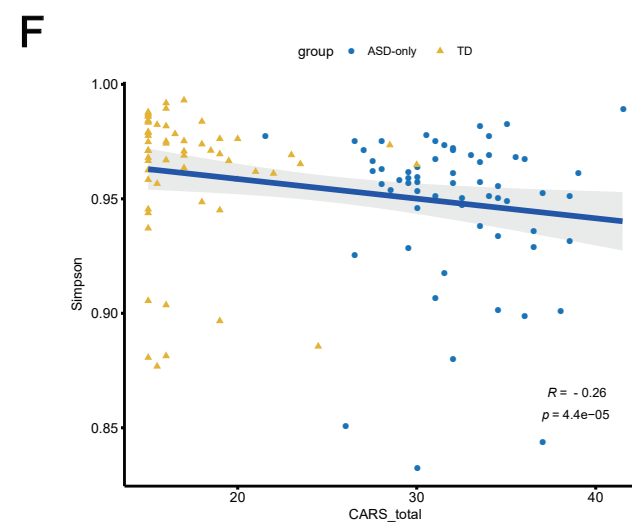

Supplement: Supplementary Figure 5 — Correlation between the richness and diversity of bacterial and clinical characteristics in selected groups. (A) Correlation in participants with comorbid DD/ID (DD/ID and ASD+DD). (B) Correlation in participants without comorbid DD/ID (TD and ASD-only). Shannon index was negatively correlated with the total CARS score in group whether combined DD/ID (C) or not (D). Total CARS score was negatively correlated with Simpson index in group whether combined DD/ID (E) or not (F). [file Data_Sheet_5.PDF]

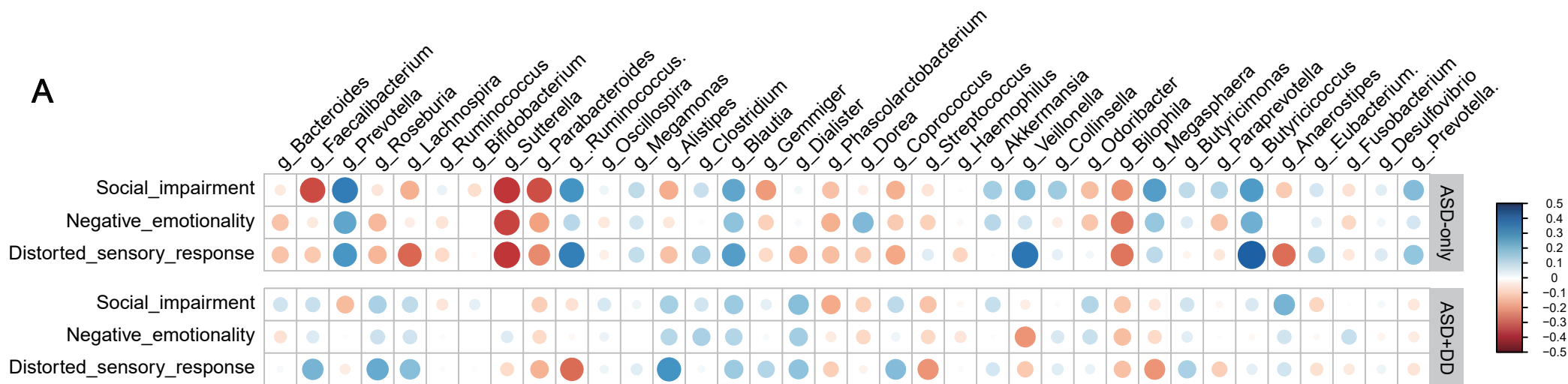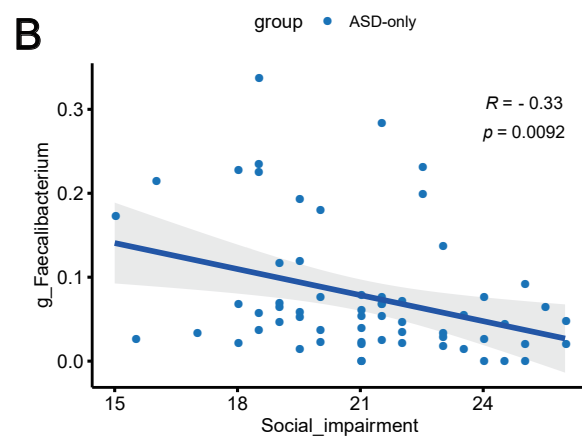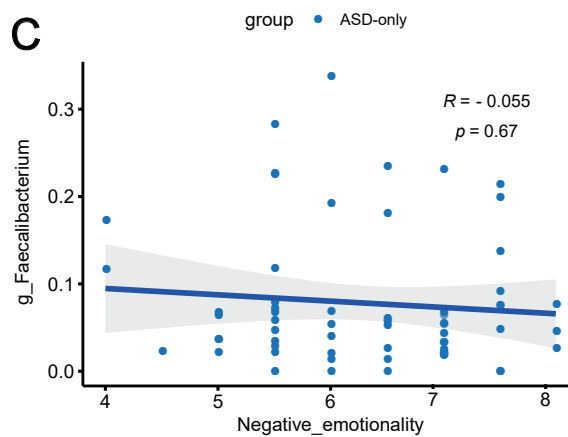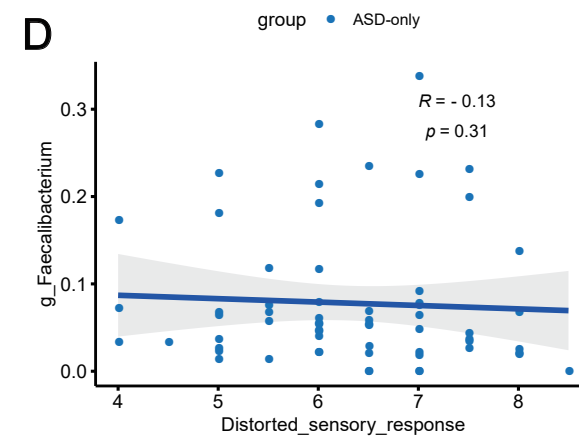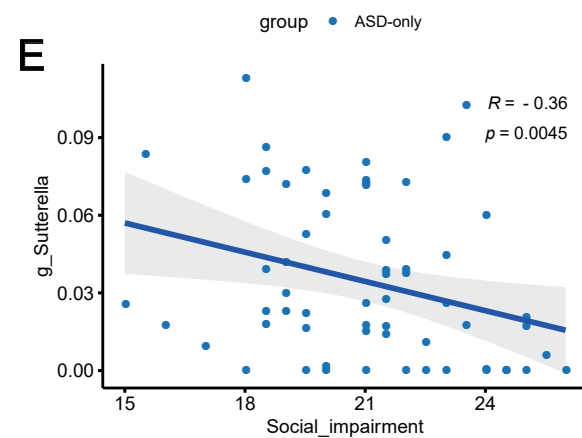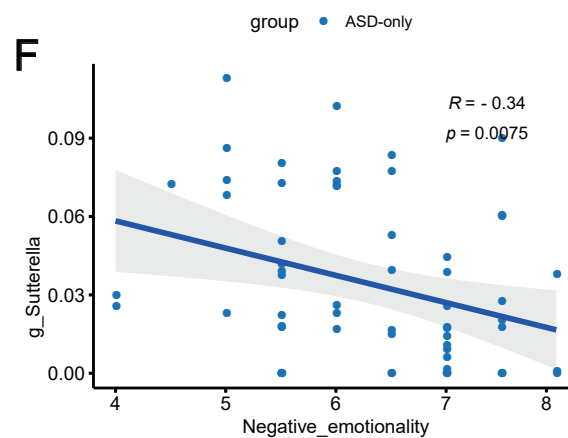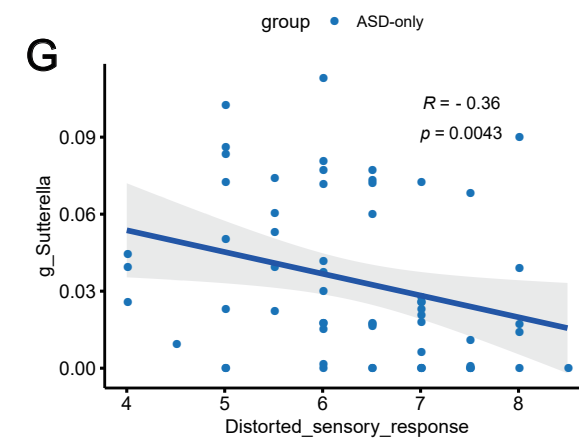

Supplement: Supplementary Figure 6 — Association between bacterial relative abundances and three primary CARS domains in subgroups of children with ASD. (A) Heatmaps showing correlations between genus and Social impairment, Negative emotionality, and Distorted sensory responses. In the ASD-only group, the abundance of Faecalibacterium was negatively correlated with social impairment (B), negative emotionality (C), and distorted sensory responses (D) in the ASD-only group. The Sutterella genus showed significant negative correlations with social impairment (E), negative emotionality (F), and distorted sensory responses (G). [file Data_Sheet_6.PDF]
